# Scheduled Telephone Support for Internet Cognitive Behavioral Therapy for Depression in Patients at Risk for Dropout: Pragmatic Randomized Controlled Trial

**DOI:** 10.2196/15732

**Published:** 2020-07-23

**Authors:** Satu Pihlaja, Jari Lahti, Jari Olavi Lipsanen, Ville Ritola, Eero-Matti Gummerus, Jan-Henry Stenberg, Grigori Joffe

**Affiliations:** 1 Department of Psychiatry University of Helsinki and Helsinki University Hospital Hospital District of Helsinki and Uusimaa Helsinki Finland; 2 Department of Psychology and Logopedics Faculty of Medicine University of Helsinki Helsinki Finland

**Keywords:** internet CBT, depression, scheduled telephone support, adherence, routine clinical practice

## Abstract

**Background:**

Therapist-supported, internet-delivered cognitive behavioral therapy (iCBT) is efficient in the treatment of depression. However, the optimal mode and intensity of therapist support remain to be identified. Scheduled telephone support (STS) may improve adherence and outcomes but, as it is time- and resource-consuming, should be reserved for patients for whom the usual support may be insufficient.

**Objective:**

This paper aims to reveal whether add-on STS for patients at risk of dropping out improves treatment adherence and symptoms in iCBT for depression.

**Methods:**

Among patients participating in an ongoing large observational routine clinical practice study of iCBT for depression delivered nationwide by Helsinki University Hospital (HUS-iCBT), those demonstrating a ≥14-day delay in initiation of treatment received invitations to this subsidiary STS study. A total of 100 consenting patients were randomly allocated to either HUS-iCBT as usual (control group, n=50) or HUS-iCBT plus add-on STS (intervention group, n=50). Proportions of those reaching midtreatment and treatment end point served as the primary outcome; secondary outcomes were change in Beck Depression Inventory (BDI)–measured depressive symptoms and time spent in treatment.

**Results:**

Add-on STS raised the proportion of patients reaching midtreatment compared with HUS-iCBT as usual (29/50, 58% vs 18/50, 36%; *P*=.045) and treatment end point (12/50, 24% vs 3/50, 6%; *P*=.02). Change in BDI score also favored add-on STS (3.63 points vs 1.1 points; *P*=.049), whereas duration of treatment did not differ.

**Conclusions:**

Add-on STS enhances adherence and symptom improvement of patients at risk of dropping out of iCBT for depression in routine clinical practice.

**Trial Registration:**

International Standard Randomised Controlled Trial Number (ISRCTN) 55123131; http://www.isrctn.com/ISRCTN55123131.

## Introduction

Despite the growing burden of mental health disorders, treatment is available for fewer than half of those in need [1]. Among mental health conditions, depressive disorders are some of the most prevalent [2]. As a leading cause of disability worldwide [3], they represent a major burden for health care systems [4].

For depressive disorders, recommended first-line treatments include psychological interventions, specifically psychotherapies [5]. Although psychotherapies are highly acceptable among clients [6,7], their availability is limited [8]. Some of the main challenges are shortage of professionals, perceived stigma, cost, and long distance to services [9].

Computer-based delivery of psychotherapy improves access to psychological treatments of depression [10]. Internet-delivered cognitive behavioral therapy (iCBT) entails modules or lessons delivering cognitive behavioral therapy (CBT) concepts via the internet [11]. iCBT for depression offers solutions to such challenges as poor treatment availability, fidelity and affordability, and constraints of location and time [10,12]. In randomized controlled trials (RCTs), iCBT for depression demonstrates efficacy equal to that of traditional face-to-face CBT but only if accompanied by therapist support [11,13,14], wherein the program involves therapist guidance, typically via email [15]. Fully automated iCBT treatments, supported by only computerized reminders, tend to be less efficacious than the ones with therapist guidance [16,17]**.**

High variation in attrition rates indicates that in some iCBT studies, a considerable number of patients fail to complete the treatment even in therapist-supported programs [18]. In a review comprising 19 studies on iCBT with therapist support for various mental disorders, the dropout (defined as termination at any point between registering for treatment and completing follow-up questionnaires) ranged from 2% to 83%, with a weighted average of 31% [19]. Two reviews of RCTs for therapist-supported iCBTs have shown a dropout rate of approximately 20% [10,20] and one meta-analysis on iCBT for depression showed a mean dropout rate of 28% [21]. However, in a large routine clinical practice study of a primary care population, 82% of participants failed to finish the program [22].

A meta-analysis of iCBT studies [19] identified several potential predictors of attrition, including age, gender, socioeconomic and relationship status, duration and target of the psychological problem, comorbidity, client-related psychological variables, treatment credibility, computer experience, motivation, the type of support medium, and quality and duration of clinician contact. Research evidence on these variables is, so far, limited. Adding fees, choice, reminders, and clinician support seems to strengthen adherence [23]. The essential elements of the contact are, however, debatable. Eysenbach [18] hypothesized that dropout is more likely the more “virtual” the support is. Synchronous telephone contact may be less virtual than is support in writing. Thus far, to our knowledge, no meta-analysis has yet directly investigated the impact of the contact medium used in iCBT for depression [24].

Telephone calls offering technical assistance facilitated self-monitoring during a web-based intervention versus only automated assistance [25]. Automated but personalized telephone prompts were useful for maintaining adherence in a study on weight control intervention [26]. However, in iCBT for depression, weekly telephone calls by a lay telephone counsellor did not confer additional advantage for callers to a helpline service [27]. The researchers proposed that more benefits might be achieved with support provided by a clinician. Nevertheless, therapist-delivered telephone and email support in iCBT for depression did not yield improvement in outcomes or in dropout rates [24]. Neither did technician-delivered telephone support demonstrate advantages over a clinician-moderated online discussion forum in iCBT for social phobia [28], although in the same program, telephone calls facilitated symptomatic improvement and adherence versus automatic reminders only [29]. In an iCBT aftercare program for bulimia nervosa, telephone prompts by a research assistant improved adherence [30], as did telephone support by a technician in iCBT for depression in primary care compared with text-based support [31]. Furthermore, in a trial of patients with treatment-resistant obsessive-compulsive disorder (OCD), scheduled telephone support (STS) reduced dropout rate compared with optional telephone support [32].

Even though STS has improved adherence, it consumes more resources than iCBT as usual and the target group therefore needs careful defining. Several potential factors predict dropout [19], but to the best of our knowledge, no clear-cut criteria for identification of increased dropout risk exist, and no trials have directly studied the effects of extra support in iCBT for depression to aid patients at such risk.

This RCT investigated whether STS added to iCBT as usual enhanced adherence to and effectiveness of iCBT for depression in a sample of patients at high risk for dropping out, (ie, those demonstrating delayed start of iCBT).

We expected that the add-on STS would enhance adherence to treatment, reduce symptoms of depression, and shorten the duration of treatment in patients at risk for dropping out.

## Methods

### HUS-iCBT as Usual

The department of psychiatry of Helsinki University Hospital (HUS) has developed and is providing nationwide a range of original Finnish-language iCBT programs (further referred to as HUS-iCBTs) for common psychiatric disorders to which all physicians licensed in Finland can refer patients. To receive HUS-iCBT for depression, patients must be diagnosed with a depressive episode (code F32-F33 in International Classification of Diseases 10) and be aged ≥18 years. Exclusion criteria are current alcohol misuse as judged by the referring physician; known diagnosis of schizophrenia or other psychotic disorder, bipolar disorder, serious personality disorder, or neurological or neuropsychiatric disorder that adversely affects the patient’s cognitive performance; or demonstrated, reported, or observed suicidal intentions. However, each referring physician holds primary judicial responsibility for overall treatment and, prior to referral, verifies the diagnosis and checks all of these criteria. No pretreatment interview by the therapy provider is thus necessary.

The HUS-iCBT for depression consists of 7 consecutive modules and is 109 pages in total, including texts, videos, illustrations, and assignments. Contents include information on depression and CBT, goal definition, behavioral activation, cognitive restructuring, advice on a balanced life, relapse prevention, and homework. Patients use a secure online identity system to enter the program. They are required to report possible suicidal thoughts. The time schedule of the treatment is flexible, although patients are prompted to progress at a pace of 1 module per week. Active engagement is required, since progress to the next module is prevented if any of the previous module assignments is not completed.

The program sends email prompts for new messages and login reminders. The same internet therapist follows each patient throughout the treatment, and they communicate asynchronously via text-based message board within the therapy program. The therapist sends messages in the beginning, at midtreatment, at the sixth module, and at the end point; patients receive encouragement to write to the therapist any time with their questions or concerns. The therapist comments on the completed tasks, offering praise and support for the patient. The patients receive automatic messages recapitulating the contents of the modules, which are distinguished from the messages sent by therapists. In addition, the patients receive an email prompt if no login occurs for 2 weeks and when they receive a new message. Automatic prompts notify therapists of new messages or in case of any sign of suicidality. When expected progress fails, the therapist tries to contact the patient by a message within 2 to 4 weeks. If the patient still does not appear in the program, the therapist tries to reach the patient by telephone (therapist-initiated telephone calls are not used for other purposes). If the patient remains unreachable by telephone, a letter on paper is sent to uncover the reason for no show.

### Study Patients and Design

#### Large Simple Observational Study

All patients referred to the HUS-iCBT for depression are invited to participate in an ongoing nationwide, low-threshold, flexible–time schedule, observational, routine clinical care study on the effectiveness of HUS-iCBT in depressive disorders (original report under preparation). The only additional criterion for that study is a signed informed consent. All consenting patients (currently 79% of those accepted for the HUS-iCBT) are eligible to participate and are enrolled into that study.

#### The STS Study

The current study was a subsidiary RCT branch of the observational study described above. Patients participating in the observational study received invitations to participate in the STS study if they had not proceeded in the HUS-iCBT for ≥14 days after their first entry, a delay interpreted as increased risk for dropping out. The patients were enrolled subsequently from September 2015 to October 2016 until the number of participants reached 100. These patients were randomly allocated to either HUS-iCBT as usual plus add-on STS (add-on STS group, n=50) or to HUS-iCBT as usual (control group, n=50) when a 14-day delay was detected, either during or after completing the first module of the treatment. The maximum time span allowed for this study participation since initiation was 6 months.

### Add-on STS Intervention

In addition to the standard HUS-iCBT, the add-on STS intervention group received 8 weekly 15-minute telephone calls, the first at the beginning of iCBT and the subsequent calls during each of the 7 modules, without any further calls regardless of patient progress. If necessary (eg, if a patient was reached in a bad moment or asked the therapist to call again), new calls were allowed, amounting to more than the intended 8 calls. During the first call, selection of individual goals took place. The tasks and themes of each module were discussed in the calls that followed each module. Support was individually tailored and followed the principles of the model of supportive accountability [33], which combines elements of motivational theory, organizational psychology, and computer-mediated communication to create a framework for supported computerized treatment. According to this model, combining elements of support and accountability with a legitimized and trustworthy relationship increases adherence. If delays occurred, the prescheduled calls were generally not replaced, although therapists had final judgement over this.

### Therapists

All 5 therapists involved in this trial were clinical psychologists employed by HUS and had at least 2 years of work experience with depressed patients. They attended a 1-day training session in HUS-iCBT on the study protocol of the above-mentioned observational study and of the current STS study, the STS methodology, and the International Council for Harmonisation Good Clinical Practice (ICH-GCP) guidelines. Their role included providing feedback on assignments and support with any patient issues. Every 2 months, they participated in a group supervision session, discussing any concerns or issues arising. At any time, the therapists could consult a clinical psychologist with advanced iCBT experience.

All 5 STS therapists were at the same time also providing regular HUS-iCBT for other patients, and in this study, each treated both add-on STS intervention and control groups. Study patients were assigned to each therapist randomly, depending on that therapist’s current workload, resulting in the 5 therapists having 35, 30, 18, 14, and 3 patients.

### Outcome Measures

Primary outcome measures were proportion of patients reaching midtreatment (third module) and end point (seventh module). Secondary outcome measures were change (from start to completion or to dropout, with last observation carried forward) in Beck Depression Inventory (BDI) [34] scores and treatment time (in days) from start to end point (or premature discontinuation). The internet-administered BDI has good psychometric properties [35]. The patients completed it online at the beginning, midtreatment, and end point.

At enrollment, all patients completed 6 demographic questions. The rest of the demographic data were collected from the referrals. The therapists recorded the number and duration of telephone calls. The experienced usefulness was recorded at the end of each module by visual analog scale, where “not at all useful” equaled 0 and “very useful” equaled 10.

### Ethics and Legislation

The study followed the ICH-GCP and Finnish national regulations. The study protocol was approved by the Ethics Committee of HUS and by pertinent institutional authorities. After reading a complete description of the study, the patients provided informed consent electronically. The trial was registered at the International Standard Randomised Controlled Trial Number (ISRCTN) registry (ISRCTN55123131).

### Statistical Analyses

Primary analyses, based on the intention-to-treat principle, included all patients. Analysis of variance and chi-square tests served to reveal group differences in baseline characteristics and in dropout rates.

Group comparisons of patients reaching midtreatment and end point employed chi-square tests. Cox regression survival analysis served to determine group differences in survival probabilities (ie, time spent in treatment before dropping out during the 6-month time period). This analysis was adjusted for experienced session usefulness at the end of the first session, measured by visual analog scale. The Mann-Whitney test served for group comparisons of change in depression (BDI scores) from baseline to last observation carried forward and treatment length (in days).

Statistical analyses employed SPSS Statistics (version 25; IBM Corp) for Windows (Microsoft Corp).

## Results

### Baseline Characteristics

**Figure 1:**
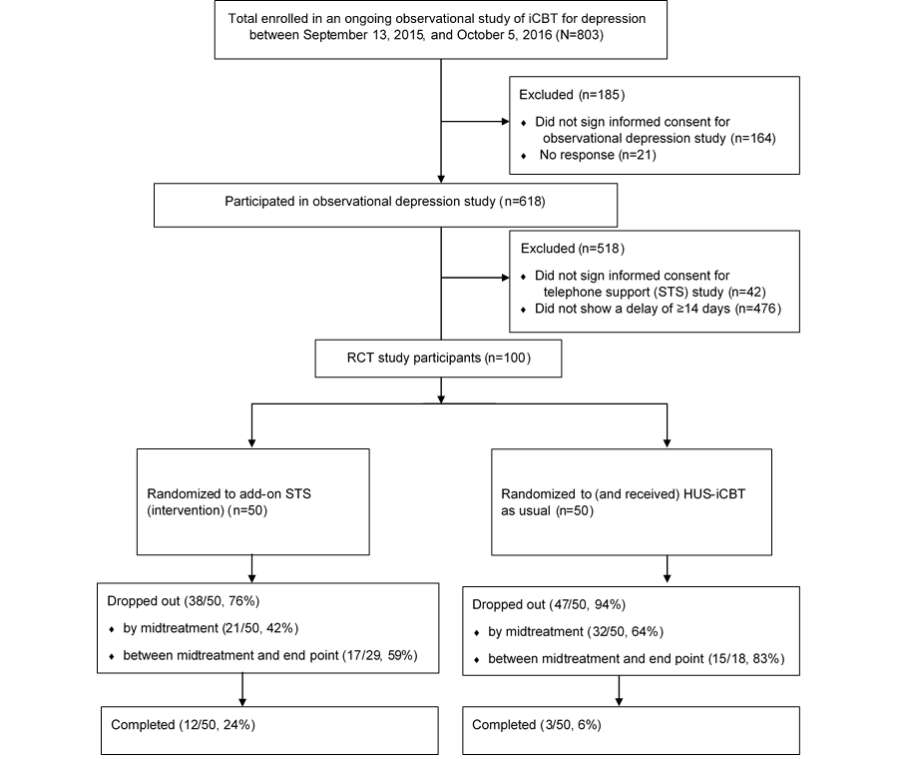
Scheduled telephone support in iCBT for depression: patient flow diagram. HUS-iCBT: Helsinki University Hospital internet-based cognitive behavioral therapy programs; iCBT: internet-delivered cognitive behavioral therapy; RCT: randomized controlled trial; STS: scheduled telephone support.

Analyses comprised 50 patients in the add-on STS group and 50 patients in the control group (see Figure 1).

Of 618 patients of the observational depression study, all 618 (100%) were invited to participate in the STS study, which amounts to 77.0% of the 803 patients enrolled during the time period between September 13, 2015, and October 5, 2016. Of the 618 invited, 576 patients (93.2%) agreed to participate. Of these 576 patients, 476 (82.6%) did not show a 14-day delay in beginning the treatment and were excluded on that criterion. A total of 6 patients of the add-on STS group never received any telephone calls (ie, did not receive the STS intervention).

No significant differences between groups emerged at baseline (see Table 1). Some patients did not provide several baseline demographic data (marital, educational, and employment statuses and use of medication or sick leave within last 6 months). However, there was no association between missing data and group membership. Interpretations of results concerning primary and secondary outcomes remained the same after controlling for an indicator of nonresponse to these demographic questions.

**Table 1:** Baseline group characteristics.

		Add-on STS^a^ (n=50)	Control (n=50)	Total (n=100)	Chi-square (*df*)	*t* test^b^ (*df*)	*P* value
Female, n (%)		34 (68.0)	32 (64.0)	66 (66.0)	0.18 (1)	N/A^c^	.67
**Referred from, n (%)**					6.50 (5)	N/A	.26
	Primary health care	28 (56.0)	23 (46.0)	51 (51.0)			
	Private health care	11 (22.0)	15 (30.0)	26 (26.0)			
	Occupational health care	7 (14.0)	2 (4.0)	9 (9.0)			
	Student health care	2 (4.0)	5 (10.0)	7 (7.0)			
	Specialized psychiatry	1 (2.0)	3 (6.0)	4 (4.0)			
	Unspecified	1 (2.0)	2 (4.0)	3 (3.0)			
**Marital status, n (%)^d^**					0.80 (3)	N/A	.85
	Married	6 (42.9)	4 (28.6)	10 (35.7)			
	Living together	2 (14.3)	2 (14.3)	4 (14.3)			
	Not married	4 (28.6)	6 (42.9)	10 (35.7)			
	Divorced	2 (14.3)	2 (14.3)	4 (14.3)			
**Educational level, n (%)^d^**					0.80 (3)	N/A	.85
	Elementary school	1 (7.1)	2 (14.3)	3 (10.7)			
	Secondary/vocational	8 (57.1)	7 (50.0)	15 (53.6)			
	College/university Bachelor	3 (21.4)	2 (14.3)	5 (17.9)			
	College/university Master	2 (14.3)	3 (21.4)	5 (17.9)			
**Employment status, n (%)^d^**					4.88 (3)	N/A	.18
	Full time	11 (78.6)	7 (50.0)	18 (64.3)			
	Part time	1 (7.1)	0 (0)	1 (3.6)			
	Unemployed	2 (14.3)	6 (42.9)	8 (28.6)			
	Retired	0 (0)	1 (7.1)	1 (3.6)			
**Medication, n (%)^d^**					0.16 (1)	N/A	.69
	None	5 (35.7)	4 (28.6)	9 (32.1)			
	Present^e^	9 (64.3)	10 (71.4)	19 (67.9)			
Sick leave within 6 months, n (%)^d^		9 (64.3)	6 (42.9)	15 (53.6)	129 (1)	N/A	.26
Age, mean (SD)		37.40 (12.16)	34.82 (10.99)	36.11 (11.10)	N/A	1.11 (98)	.27
BDI^f^ at baseline, mean (SD)		21.49 (7.15)	23.33 (10.04)	22.41 (8.75)	N/A	1.04 (96)	.30

^a^STS: scheduled telephone support.

^b^2-tailed *t* test.

^c^N/A: not applicable.

^d^Information available for 14 patients in add-on STS intervention group and for 14 control group patients.

^e^Anxiolytic or antidepressant.

^f^BDI: Beck Depression Inventory.

Since there was a considerable amount of missing data in some of the baseline characteristics, we performed comparisons (using a 2-tailed *t* test) of depression at baseline (BDI score) between patients that filled in (n=28) and did not fill in (n=72) a separate questionnaire of demographics. There was no difference in depression at baseline between the groups (t_96_=0.445; *P*=.66).

No adverse events, such as hospitalization or serious illness, occurred during the study. Altogether, 44 of the 50 (88%) patients in the add-on STS intervention group received the previously scheduled telephone calls (versus a total of 5 patients received optional calls in the control group), as seen in Table 2.

Patients in the add-on STS group reached an average of 3.54 modules, while patients in the control group reached an average of 2.46 modules (see Table 3).

**Table 2:** Mean number and duration of telephone calls received.

Telephone call characteristics	Add-on STS^a^ (n=50)	Control (n=50)
Proportion of patients who received calls, n (%)^b^	44 (88)	5 (10)
Number of calls per patient, mean (SD), range^b^	4.63 (3.58), 0-11	0.32 (0.99), 0-5
Average duration of calls (minutes), mean (SD)^b^	13.09 (5.25)	6.20 (2.17)
Cumulative duration of calls (minutes), mean (SD), range^b^	73.37 (48.95), 5-165	1.95 (6.11), 0-30
Assessed average therapist time (minutes), n^c^	132	55

^a^STS: scheduled telephone support.

^b^Missing values for 9 patients in each group.

^c^In addition to contact time itself, STS takes approximately 4 more minutes for preparation of the call and additional documentation, resulting in a total of 77 additional minutes for STS intervention vs HUS-iCBT as usual.

**Table 3:** Module reached by patients in each group during 6 months.

	Module reached, n (%)								Last module reached, mean (SD)
	0	1	2	3	4	5	6	7	
Add-on STS^a^	50 (100)	45 (90)	33 (66)	28 (56)	26 (52)	19 (38)	13 (26)	13 (26)	3.54 (2.57)
Control	50 (100)	48 (96)	27 (54)	19 (38)	15 (30)	7 (14)	4 (8)	3 (6)	2.46 (1.88)

^a^STS: scheduled telephone support.

### Outcomes

#### Adherence (Primary Outcome)

**Figure 2:**
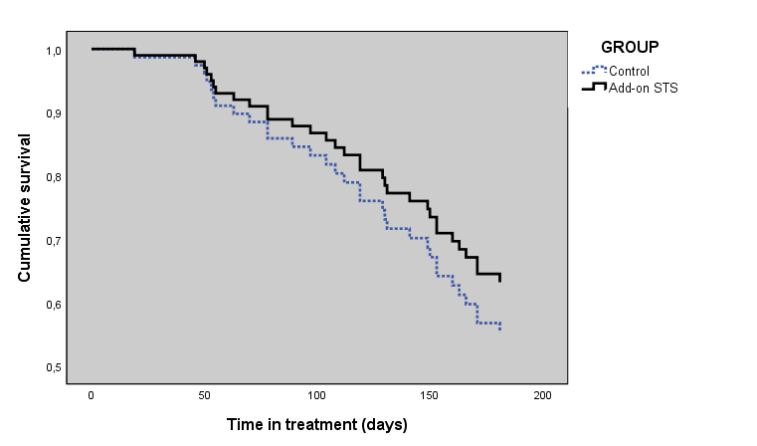
Cox regression survival curves for staying in treatment for the add-on STS and control groups at 6 months. STS: scheduled telephone support.

Of the 100 randomized patients, 21 of the 50 patients in the add-on STS group (42%) and 32 of the 50 patients in the control group (64%) dropped out by midtreatment. Corresponding figures for the end point were 38 of 50 patients (76%) and 47 of 50 patients (94%). The proportion who reached midtreatment and end point favored the add-on STS group significantly (Table 4). Cox regression survival analysis revealed no difference in the timing of dropout between groups (survival curves χ²_2_=2.5, *P*=.48; hazard ratio=1.29, 95% CI 0.64-2.61, *P*=0.48), as shown in Figure 2.

#### Secondary Outcomes

The BDI-measured change from baseline showed a statistically significant difference also favoring the add-on STS group. This result also remained when analyses were carried out using linear mixed models, which handles missing values more efficiently and without imputing the missing values (*F*_2,30_=3.77; *P*=.04). No statistically significant differences emerged for days spent on therapy (*P*=.67).

**Table 4:** Effects of add-on STS on adherence, depression, and time in treatment.

Outcome		Add-on STS^a^ (n=50)	Control (n=50)	Chi-square (*df*)	Mann-Whitney test	*P* value
Reached midtreatment, n (%)^b^		29 (58)	18 (36)	4.9 (1)	N/A	.045^c^
Completed the program, n (%)^b^		12 (24)	3 (6)	6.4 (1)	N/A	.02^c^
**Change in BDI^d^ from baseline^c,e^**				N/A^f^	1455.5	.049^c^
	Change in BDI from baseline, mean (SD)	3.63 (5.94)	1.06 (4.82)			
	Change in BDI from baseline, median (P25;P75)^g^	0 (0.0;8.5)	0 (0.0;1.0)			
**Treatment time in days^e^**				N/A	1190.5	.67
	Treatment time (days), mean (SD)	136.61 (52.18)	141.36 (48.08)			
	Treatment time (days), median (P25;P75)	166.95 (96.27;183.00)	161.5 (100.25;183.00)			

^a^STS: scheduled telephone support.

^b^Primary outcome.

^c^Last observation carried forward.

^d^BDI: Beck Depression Inventory.

^e^Secondary outcome.

^f^N/A: not applicable.

^g^25th and 75th percentiles.

## Discussion

### Principal Results

In patients at risk of dropout during iCBT for depression, add-on STS yielded an increased proportion of patients reaching midtreatment (29/50, 58% vs 18/50, 36%; *P*=.045) and proportion of patients who completed treatment (12/50, 24% vs 3/50, 6%; *P*=.02) compared with the control group. Moreover, depressive symptoms decreased more for those with add-on STS than with HUS-iCBT alone (mean change 3.63 vs 1.06; *P*=.049), but the survival analysis did not reveal any statistically significant differences in survival probabilities, that is, time spent in treatment before dropping out (*P*=.67).

### Comparison With Prior Work

Two other RCTs on STS have reported positive results. Notably, both studies used iCBT with no text-based support, meaning that comparison of our results with these previous reports should be undertaken with caution.

Gilbody and coauthors [31] compared STS to iCBT with no STS in primary care patients with depression, reporting improvement in symptom change and a decrease in dropout rate from 71% to 54% at midtreatment and from 90% to 81% at end point in their STS group patients compared with their control group patients with access only to a support helpline. Despite the at-risk population in the current study, our figures seem comparable (drop from 32/50, 64% to 21/50, 42% at midtreatment and from 48/50, 96% to 38/50, 76% at end point). Kenwright and colleagues [32], in their iCBT trial comparing STS to patient-initiated calls, reported an STS-induced change from 59% to 14% for early dropout rate and from 64% to 50% for late dropout rate. Their patients, however, suffered not from depression but from OCD. OCD is known to often be difficult to treat and, as a group, patients in that study could thus also be counted as being at risk of dropping out. Nevertheless, due to the differences in population and methodology, the results cannot be directly compared with ours.

Of note, completion of the program does not always indicate treatment success in iCBTs. For instance, in iCBT for depression, there seems to exist a subgroup of patients who benefit rapidly and therefore prematurely discontinue the treatment because it is subjectively perceived as unneeded [36]. In our study, in-depth analysis of reasons and exact time point of dropout could not be performed due to having only 3 measurement points of depression.

Not all RCTs on STS showed the desired results, however. In one depression study [24], patients received either email or STS support added to iCBT, but with no differences in dropout rates. Similarly, adherence did not improve with STS added to email in a headache study by Andersson et al [37], nor did STS demonstrate advantages over a clinician-moderated online discussion forum in terms of adherence in an RCT on social phobia from Titov and colleagues [38]. Interestingly enough, all these negative studies included only unselected, self-referred participants. In contrast, we focused only on physician-referred patients (51/100, 51% of them from primary care) whose initiation of iCBT was delayed by ≥2 weeks, which was considered a risk of dropping out. These patients may have experienced a special challenge in terms of adherence, since unlike their counterparts in the majority of previous RCTs, they may not always have originally been motivated to use iCBT; they had no contact with a therapist or other research personnel at screening (a contact that might have improved adherence), and they showed a possible lack of engagement at the beginning of iCBT.

The education level of supporting personnel may also matter, but data on this issue are conflicting. In the negative-result depression study by Lindner and colleagues [24], support providers were psychology students, whereas our positive results were achieved with support personnel being certified, experienced clinical psychologists. Nevertheless, positive results also emerged in the depression study of Gilbody and colleagues [31], in which contact personnel comprised telephone support workers. Moreover, STS personnel involved in other (nondepression) conditions differed. Therapists were the support providers in one positive-result OCD trial [32] and in one negative headache trial [37], whereas technicians took this role in the negative-result social phobia trial of Titov and colleagues [38].

In psychotherapy research, researcher allegiance, a tendency to favor certain preferred treatments, is widely discussed [39]. Treatment developers’ trials tend to yield better outcomes than the later research performed by others, probably due to researcher allegiance. One alternative explanation is that developers themselves gain an advantage through their intense clinical and research involvement with the treatment; they become superior in treatment delivery [40]. Gilbody and colleagues [31] attributed their poor outcomes and adherence—results inferior to those demonstrated by treatment developers—to differing means of service provision. Gilbody and his group performed their trial within the Randomised Evaluation of the Effectiveness and Acceptability of Computerised Therapy trial, a large National Health Service–funded program in which treatment provision was decentralized. In contrast, in our setting, the developer of the HUS-iCBT program, which was the Department of Psychiatry of Helsinki University Hospital, was the only nationwide centralized provider of both iCBT and the add-on STS. This may explain our somewhat better-than-expected results.

The mechanism of the desirable effects of STS is uncertain. These effects may result from increased therapist contact time, from the telephone as the medium versus written contact only, or from the proactivity of therapist-initiated scheduled contact.

What has been maintained is that increased therapist contact time in iCBT for mood and anxiety disorders correlates strongly with treatment outcome [41]. This association has not always been detectable in reviews, however [24], and has been considered true only up to a certain threshold [42]. Gilbody and colleagues [31] did not describe their average STS time. The STS time in the iCBT study on OCD [32] was 232 minutes versus 178 minutes for the optional contact, which was markedly more than the typical 90 to 150 minutes per patient in iCBT trials [43]. In our study, average added telephone contact time for the add-on STS intervention group was 73 minutes per patient (versus 2 minutes for the control group). Given the average 55-minute therapist time per patient for HUS-iCBT, SPS means a 2.4-fold increase in contact time. Neither the study by Kenwright and colleagues [32] or by Gilbody and colleagues [31] nor the present one has been a dose-finding trial. We were unable to locate any dose-finding trials on the effects of contact time in added STS in any iCBT program. It therefore remains unclear whether those 54 additional minutes of STS time for OCD or our 73 additional minutes for depression are optimal in terms of adherence or symptomatic improvement in iCBT. Nevertheless, the 2.4-fold increase in resource allocation when weighted against health benefits achieved may be of interest to administrative decision makers.

The telephone as a medium is one of the synchronous contact modes, in contrast to asynchronous contact via written message (usually referred to as minimal contact) in the majority of iCBT studies. Furthermore, unlike text message, a telephone conversation conveys nonverbal voice signals (such as intonation and pausation). Since in these 2 studies [31,32] the control groups received no text-based support, no conclusions can be drawn on the specific role of the telephone as a medium. Moreover, other RCTs comparing telephone support with email support found no differences in treatment outcomes or dropout rates [24,37,38]. The role of the voice contact in iCBTs and the specific mechanisms of action therein remain poorly understood.

### Limitations and Strengths

Our sample size was relatively small. Although between-group differences were statistically significant, statistical power was insufficient for subgroup analyses and hence for identification of a subpopulation for optimal therapist resource allocation.

Due to selection of population (patients at risk for dropout in everyday clinical practice), the dropout rate in our study was expectedly high, with 76% (38/50) in the STS group and 94% (47/50) in the control group dropping out, when dropout is defined as those who did not finish the treatment in 6 months. In addition, the patients did not have a strict deadline of 6 months, even though the measurements were conducted at that point. Studies comparing iCBT programs with unselected populations reported a 62% dropout rate in everyday clinical practice and 15% to 25% in RCTs [29]. Our findings underline the difficulties of maintaining adherence in routine clinical care for digital health products that demonstrate impressive results in clinical trials [44]. Nevertheless, the STS in this study showed results comparable to an 82% dropout rate in earlier observational studies on iCBT for depression with unselected population [22].

Therapist effects in our study could not be ruled out, since 2 of altogether 5 therapists treated 63 of the 100 (63%) patients. This small number of therapists is, however, too small for reliable assessment of possible therapist effects [45].

The effect of support may depend on the patient group [46]. We defined the ≥14-day delay prior to the beginning of iCBT treatment as a criterion of dropout risk based on clinical experience, since, to the best of our knowledge, no clear-cut criteria for such risk have yet been defined. This criterion might fit only a certain subgroup of our patients, meaning that the add-on STS intervention was applied for no reason also to a not-at-risk subgroup. Understanding the reasons for patient delay could help to sharpen this criterion, but in our study, data as to the reasons were unavailable.

The HUS-iCBT with add-on STS required 77 minutes more therapist time than did the HUS-iCBT as usual. Cost-benefit analysis of STS was not a subject of this study, so our results are insufficient to inform decision makers on whether to use STS for patients at risk for dropout in routine clinical practice, not to mention for patients at no such risk.

### Future Research

Future research should explore in depth possible criteria for those at risk for dropping out to discover optimal contact time with larger populations. Add-on STS in other populations, such as depressed patients who demonstrate no delay in initiation of iCBT or patients with disorders other than depression, is worth studying. Reasons for the delayed initiation of iCBT as well as other predictors of poor adherence call for exploration. Cost-benefit analysis could optimize the use of add-on STS. Add-on STS provided by professional groups of a lower educational level than that of our clinical psychologists also demands exploration.

Our study employed STS added to text-based support, but STS alone should also be a focus of research in populations at risk of dropout to reveal the unconfounded effects of STS as such. Comparison of STS with other support media, such as voice messages (asynchronous contact mode but enriched with nonverbal voice modulations) or chat (synchronous contact but devoid of nonverbal voice modulations), can yield new insights into both the mechanism of action of STS and the practical means to optimize cost benefits of support.

Possible therapist effects should be explored in further iCBT studies with a greater number of therapists with equal caseloads providing STS.

### Conclusion

STS added to the usual iCBT appears to improve both adherence and clinical symptomatology in patients with depression at risk for dropout from iCBT, but more research is required to optimize its use.

## Appendix

Multimedia Appendix 1 CONSORT-EHEALTH checklist (V. 1. 6. 1).

## Abbreviations

BDI: Beck Depression Inventory
CBT: cognitive behavioral therapy
HUS: Helsinki University Hospital
HUS-iCBT: Helsinki University Hospital internet-based cognitive behavioral therapy programs
iCBT: internet-based cognitive behavioral therapy
ICH-GCP: International Council for Harmonisation Good Clinical Practice
ISRCTN: International Standard Randomised Controlled Trial Number
OCD: obsessive-compulsive disorder
RCT: randomized controlled trial
STS: scheduled telephone support
